# Digital Approach to Rotational Speed Measurement Using an Electrostatic Sensor

**DOI:** 10.3390/s19112540

**Published:** 2019-06-04

**Authors:** Lin Li, Hongli Hu, Yong Qin, Kaihao Tang

**Affiliations:** 1 State Key Laboratory of Power Equipment and Electrical Insulation, Xi’an Jiaotong University, Xi’an 710049, China; ll123xjtu.edu.cn@stu.xjtu.edu.cn (L.L.); mrerr07@stu.xjtu.edu.cn (K.T.); 2 State Key Laboratory of Rail Traffic Control and Safety, Beijing Jiaotong University, Beijing 100044, China; yqin@bjtu.edu.cn

**Keywords:** electrostatic sensor, digital approach, rotational speed, correlation algorithm

## Abstract

In industrial production processes, rotational speed is a key parameter for equipment condition monitoring and fault diagnosis. To achieve rotational speed measurement of rotational equipment under a condition of high temperature and heavy dust, this article proposes a digital approach using an electrostatic sensor. The proposed method utilizes a strip of a predetermined material stuck on the rotational shaft which will accumulate a charge because of the relative motion with the air. Then an electrostatic sensor mounted near the strip is employed to obtain the fluctuating signal related to the rotation of the charged strip. Via a signal conversion circuit, a square wave, the frequency of which equals that of the rotation shaft can be obtained. Having the square wave, the *M/T* method and *T* method are adopted to work out the rotational speed. Experiments were conducted on a laboratory-scale test rig to compare the proposed method with the auto-correlation method. The largest relative errors of the auto-correlation method with the sampling rate of 2 ksps, 5 ksps are 3.2% and 1.3%, respectively. The relative errors using digital approaches are both within ±4‰. The linearity of the digital approach combined with the *M/T* method or *T* method is also superior to that of the auto-correlation method. The performance of the standard deviations and response speed was also compared and analyzed to show the priority of the digital approach.

## 1. Introduction

In industrial applications, rotational speed measurement is a crucial part for condition monitoring, speed control, and protective supervision of rotation equipment, such as generators, steam turbines, and gas turbines. Various kinds of tachometers based on different mechanisms, such as optical, electrical, and magnetic induction, have been developed and widely used to measure the rotational speed of target objects. W.H. Yeh presented a high-resolution optical shaft encoder to monitor the rotation behavior of a motor [1] and J. N. Lygouras presented a solution for processing the pulses from an optical encoder attached to a motor shaft [2]. W. Lord and R.B. Chatto provided a homopolar tachogenerator with low inertia and noise generation, making it particularly suitable for velocity-control systems using high-performance DC motors as the power actuators [3]. C. Giebeler designed a contactless sensor based on the giant magneto-resistance (GMR) effect for position detection and speed sensing [4]. Z. Shi implemented a tachometer using a magnetoelectric composite as a magnetic field sensor which was mounted where the magnetoelectric composites had the highest sensitivity [5]. Considering the operating mode, the rotational speed measurement method can be classified into digital and analog categories. In the analog tachometer output a voltage or current signal proportional to the speed can be used to provide a feedback signal in a closed-loop speed control system [6]. Digital tachometers have been used over the years, which utilize electronic circuits to measure an average frequency of incoming pulses from an encoder mounted on a shaft [7].

In order to overcome the hash condition, such as a high temperature, heavy dust environment, the electrostatic method has been used to realize the rotational speed measurement. The electrostatic sensor is adaptable for the speed measurement in various industrial conditions for the advantages of contactless measurement, low cost, simple structure, and easy installation and maintenance. Recently, Y. Yan and L.J. Wang utilized electrostatic sensors and a correlation algorithm to calculate the period or elapsed time and successfully obtained the rotational speed of a rotational shaft [8,9]. The electrostatic method to measure rotational speed utilizes the electrode to induce the electric field generated by carried charges on the shaft. When two materials are touched or rubbed together electrical charge is usually transferred from one to the other [10]. According to the theory of tribo-electric charging, each material has its own surface work function. Then the surface electron transfer will occur in the driven Fermi energy level [11]. The material type determines the work function, which indicates the capability of a material to hold onto its free electrons. Thus, the polarity and quantity of the charge generated due to the triboelectric friction are mainly decided by the material type and surface roughness, and are also affected by its surrounding environment, like temperature and humidity. Thus, if the shaft has a greater work function than the air, the relative motion between the rotational shaft and the air will generate some charge on the surface of shaft due to triboelectric friction. If one of the materials is a good insulator, the charge persists on its surface for a long time, and the effects of the charge transfer are readily apparent [10].

**Figure 1:**
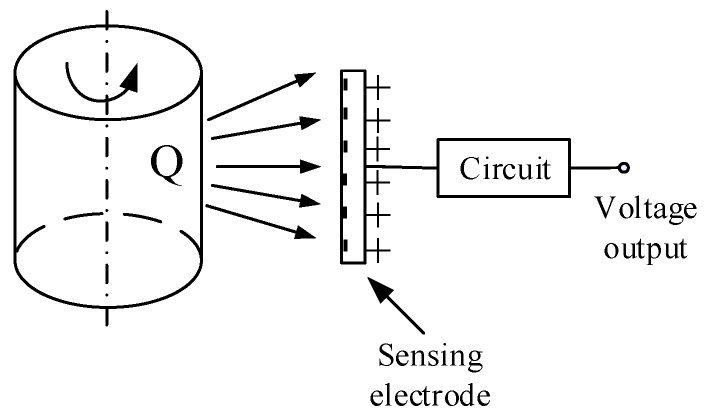
Measurement principle using the electrostatic method.

The principle of rotational speed measurement using an electrostatic sensor is shown in Figure 1. According to electrostatic induction theory, when the surface of the shaft carries some charge due to triboelectric friction with the air, it will influence the electrostatic field of its surroundings, thus, the induced charge will be generated on the surface of sensing electrode when it is installed near the shaft. The fluctuation of the induced charge on the electrode generates a current which can be converted into a voltage signal via a current-to-voltage conversion circuit. Additionally, a charge amplifier circuit can be adopted to translate the charge into a voltage signal [12]. The voltage signal collected from the electrode contains a wealth of rotational information, thus processing and analyzing the output signals from the sensor will result in obtaining further information.

**Figure 2:**
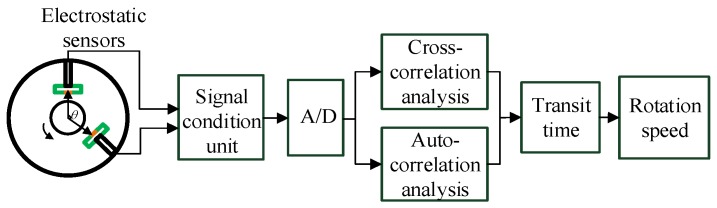
Principle of the rotational speed measurement using electrostatic sensors.

By now, electrostatic sensors in conjunction with correlation methods, including the cross-correlation method using dual electrostatic sensors and the auto-correlation method using a single electrostatic sensor, have been used to determine rotational speed [8,9,13]. Figure 2 describes the rotational speed measurement system which uses electrostatic sensors and the correlation method. Two or more channels of sensors and the corresponding condition units are connected to an A/D converter. Then a microprocessor system or a computer is needed to execute the correlation algorithm.

In the time domain, the definition of the cross-correlation function between real power signals *x*(*t*) and *y*(*t*) is:

(1) $$R_{xy}(\tau )=\underset{T\to \infty }{\lim}\frac{1}{T}\int_{-T/2}^{T/2}x(t)y(t+\tau )dt$$

**Figure 3:**
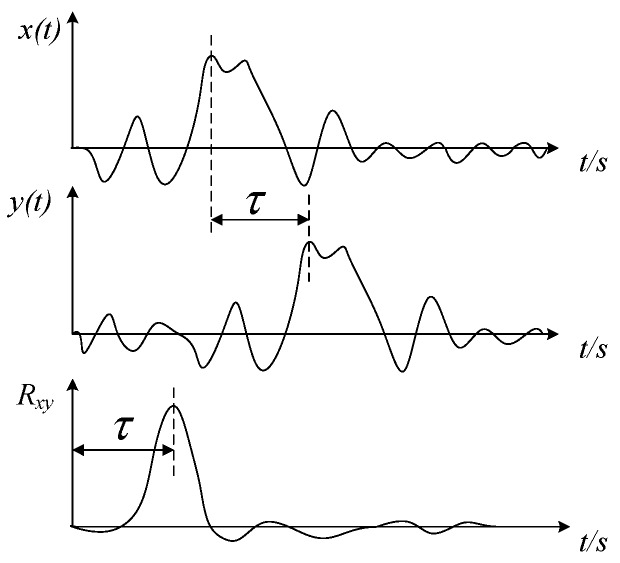
Illustration of the cross-correlation method.

Figure 3 illustrates how to obtain the time-delay between two electrostatic signals using the cross-correlation method. The rotational speed *v_c_* (revolutions per minute, rpm) can be calculated by the sensor angle spacing θ (degree) and the transit time τ (*s*):

(2) $$v_{c}=\frac{1}{T}\times 60=\frac{1}{\frac{360}{\theta }\cdot \tau }\times 60=\frac{\theta }{6\tau }$$

If *x*(*t*) and *y*(*t*) are the same signals obtained by one electrode, Equation (1) turns out to be the auto-correlation function. With respect to the auto-correlation method, only one channel of the electrostatic signal is needed. The time-delay *τ* between signal *x*(*t*) and signal *y*(*t*) is the rotational period *T*(*s*) and θ equals 360 degree. Using the auto-correlation algorithm can extract the time of rotation period. After that, the rotational speed *v_a_* (rpm) can be obtained as follows:

(3) $$v_{a}=\frac{60}{T}$$

Obviously, the correlation method needs to locate the coordinate of the first dominant peak in the waveform of the correlation function, which is influenced by the sampling rate to a great extent. At the same time, the waveforms collected by inducing the signal from a cylinder dielectric sleeve contain complex information and a faint sign of the periodical component. Although the correlation calculation of the waveform has good performance and successfully extracts the elapsed time, the computational accuracy of the correlation method is obviously affected by the sampling rate and signal noise [14].

For the sake of improving the performance of rotational speed measurement via the electrostatic method, this paper proposes an approach to generate a square wave from an electrostatic sensor in order to obtain the rotational speed via digital methods, thus eliminating the influence of the sampling rate and signal noise, and also simplifying the system complexity. In the following article, “square wave” refers to the output waveform from the comparison circuit which generates a pulse every rotational period. Implementation of a rotational speed measurement system based on this method is presented. Compared to the rotational speed measurement method using an electrostatic sensor in conjunction with correlation, this designation leaves out the AD converter and simplifies the computation code which is more adaptive for the implementation in a microprocessor system.

## 2. Measurement Principle and Finite Element Simulation

### 2.1. Measurement Principle

**Figure 4:**
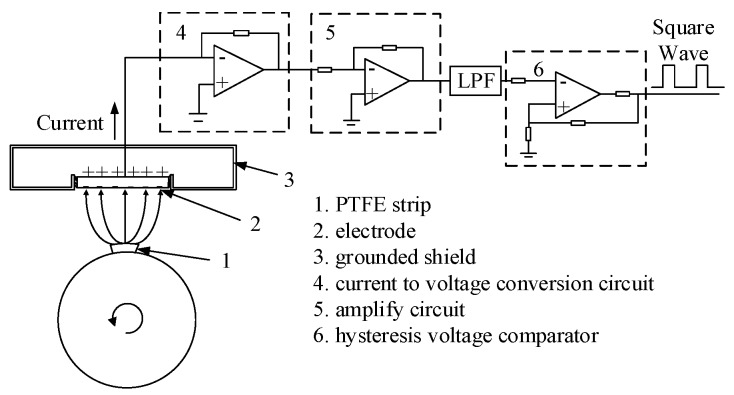
Measurement principle.

Inspired by the photoelectric method which fixes a strip of a reflection element, this experiment uses a strip of polytetrafluoroethylene (PTFE) stuck to the rotational shaft. The measurement principle is shown in Figure 4. Adopting this designation, the charge generated on the PTFE by the relative rotation with the air will pass the sensor once a revolution, which makes the waveform have a strong sign of periodicity. The electrostatic signal is firstly transformed into a voltage signal. Then, after amplifying, filtering, and comparison, the analog signal will be transformed into a square wave, which is convenient to be connected to a DSP or FPGA system to execute the following rotational speed calculation algorithm.

### 2.2. Rotation Speed Computation Algorithm

**Figure 5:**
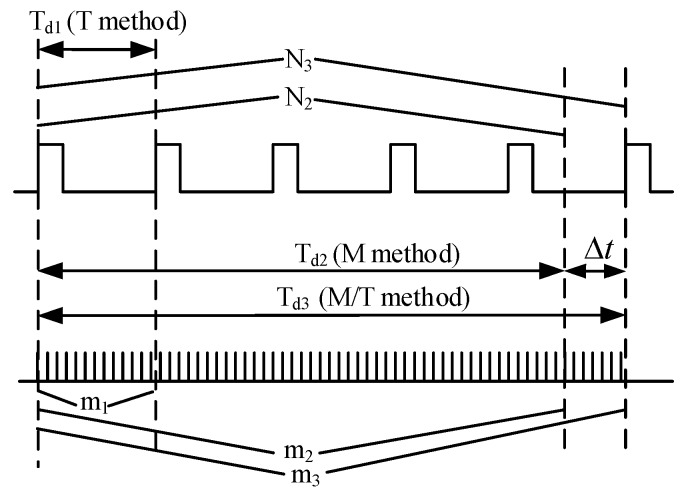
Principles of *T*, *M*, and *M/T* methods.

Usually, three methods are adopted to evaluate the speed based on these square waves: (1) Measuring the elapsed time, commonly termed as the *T* method, which calculates the reciprocal of the duration between consecutive pulses to obtain the frequency; (2) pulse counting, commonly termed as the *M* method, which counts the number of pulses generated within a prescribed period of time; and (3) constant elapsed time, commonly termed as the *M/T* method, is a combination of pulse counting and measuring elapsed time [15,16,17]. The principles of the three methods are shown in Figure 5.

As seen from Figure 5, the detecting time of the *T* method and *M* method can be obtained according the Equations (4) and (5), correspondingly, where *m*_1_ is the number of clock pulse counting during one period of square wave, *m*_2_ is the number of pulse counting during the prescribed time, and *f_s_* is the frequency of clock pulse used for counting and timing:

(4) $$T_{d1}=\frac{m_{1}}{f_{s}}$$

(5) $$T_{d2}=\frac{m_{2}}{f_{s}}$$

Then the rotational speed *v*_1_ (rpm) of the *T* method can be calculated according to Equation (6). The rotational speed *v*_2_ (rpm) of the *M* method can be calculated according to Equation (7), where *N*_2_ is the integer number of square wave during the prescribed time:

(6) $$v_{1}=\frac{60fs}{m_{1}}$$

(7) $$v_{2}=\frac{60fsN_{2}}{m_{2}}$$

Different from the *M* method, which ceases the pulse counting once the prescribed time runs out, the *M/T* category goes on counting after the prescribed time and stops at the first pulse of rotation after the prescribed time. Thus, the detecting time of the *M/T* method *T_d_*_3_ (*s*) equals *T_d_*_2_ (*s*) plus Δ*t*. Parameter *N*_3_ is the number of square waves during the detecting time. Parameter *m*_3_ is the number of clock pulses during the detecting time. The detecting time *T_d_*_3_ (*s*) can also be obtained using parameter *m*_2_ and the frequency of clock pulse *f_s_* used for timing, as shown in Equation (8). Thus, the rotational speed of the *M/T* method is calculated by Equation (9):

(8) $$T_{d3}=\frac{m_{3}}{f_{s}}$$

(9) $$v_{3}=\frac{60f_{s}N_{3}}{m_{3}}$$

The calculation errors of the three method can be derived according to Equations (10)–(12). Parameters $m_{1}^{0}$, $m_{2}^{0}$, and $m_{3}^{0}$ are the ideal pulse numbers needed to perfectly overlap the detecting time. Parameter $N_{2}^{0}$ equals the detecting time divided by the rotation period, which is not an integer in most cases.

(10) $$e_{1}=(\frac{60f_{s}}{m_{1}}-\frac{60f_{s}}{m_{1}^{0}})/\frac{60f_{s}}{m_{1}^{0}}=\frac{m_{1}^{0}-m_{1}}{m_{1}}$$

(11) $$e_{2}=(\frac{60fsN_{2}}{m_{2}}-\frac{60fsN_{2}^{0}}{m_{2}^{0}})/\frac{60fsN_{2}^{0}}{m_{2}^{0}}=\frac{m_{2}^{0}N_{2}/N_{2}^{0}-m_{2}}{m_{2}}$$

(12) $$e_{3}=(\frac{60f_{s}N_{3}}{m_{3}}-\frac{60f_{s}N_{3}^{0}}{m_{3}^{0}})/\frac{60f_{s}N_{3}^{0}}{m_{3}^{0}}=\frac{m_{3}^{0}-m_{3}}{m_{3}}$$

As seen from Equation (10), it can be observed that the error of the *T* method is low at a high speed (*m*_1_ decreases) and the *M* method resolution is not high at a low speed (*N*_2_ is not stable). However, considering the frequency of the clock pulse used for timing in this article is 150 MHz, which is significantly greater than the frequency of rotation, the counting errors of the *T* method and *M/T* method are extremely small compared to their denominators. The *T* method and *M/T* method have absolutely accurate counting numbers of the square wave from circuits 1 and *N*_3_, correspondingly. The calculating errors of the *T* method and *M/T* method mainly result from the counting number of the pulse clock ($m_{1}^{0}-m_{1}$, $m_{3}^{0}-m_{3}$). By contrast, regarding the *M* method, the difference between square counting *N*_2_ and $N_{2}^{0}$ may result in an obvious error.

The response speed can also be determined from the principle. Among them, the *T* method has the fastest response speed, which enables outputting a result every period. The *M/T* method and *M* method generate a result based on the statistical average principle, which gives them a relatively slow response speed. In summary, the *T* method is more adaptive for the dynamic measurement of variable rotational speed and the *M/T* method or *M* method is more adaptive for constant speed measurement or mean values of a certain time. Considering the response time and accuracy simultaneously, this article utilizes the *M/T* method and the *T* method to deal with the square wave from the measurement circuits.

### 2.3. Finite Element Simulation

**Figure 6:**
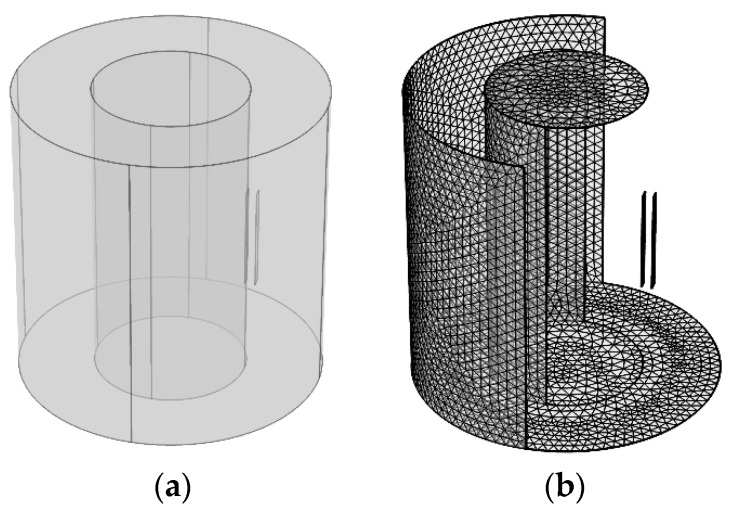
(**a**) Structure of the model, and (**b**) a mesh of the simulation model.

**Figure 7:**
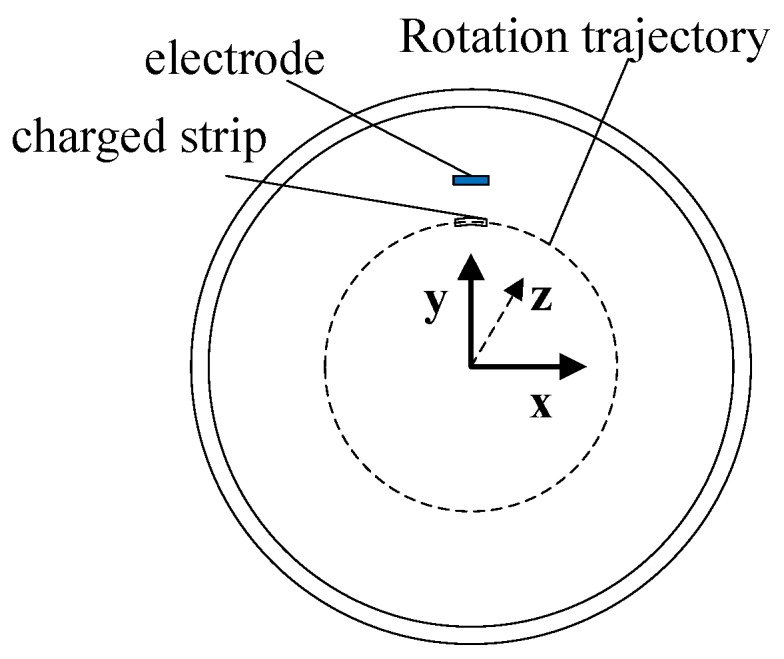
Simplified schematic.

A simulation using a strip object with evenly distributed charge was conducted utilizing COMSOL software (the COMSOL Group in Stockholm, Sweden) to imitate how the rotationally charged strip influences the induced charge on the electrostatic sensor. The model is shown in Figure 6 and the simplified two-dimensional schematic of the simulation is illustrated in Figure 7. The strip object is a 7.64 degree arc with a radius of 15 mm, which is placed tightly to the surface of the metal shaft. The length of the strip is 20 mm. The radii of metal shaft and outer shielding are 15 mm and 30 mm, correspondingly. The charge is evenly distributed on the surface of the object using the Surface Charge Density setting in COMSOL. The surface charge density is set to be 0.025 C/m^2^, so the total amount of charge on the strip is 1 μC. An electrode 20 mm long and 2 mm wide is placed 17 mm away from the central axis with the same *z* coordinate of strip. In the simulation, the strip rotates around the central axis by controlling the angle with respect to the positive axis *x*, beginning at −180 degree and stopping at 180 degree with a step size of one degree. The electrostatic field can be described by the Poisson equation and its corresponding boundary conditions:

(13) $$\left\{ \begin{array}{l} -\nabla \cdot (\varepsilon_{0}\varepsilon_{r}\nabla \varphi )=\rho \\ V_{E}=0,V_{B}=0 \end{array}\right.$$

where *V_E_* is the potential of electrode, *V_B_* is the potential of the shielding, and ρ is the space charge density.

**Figure 8:**
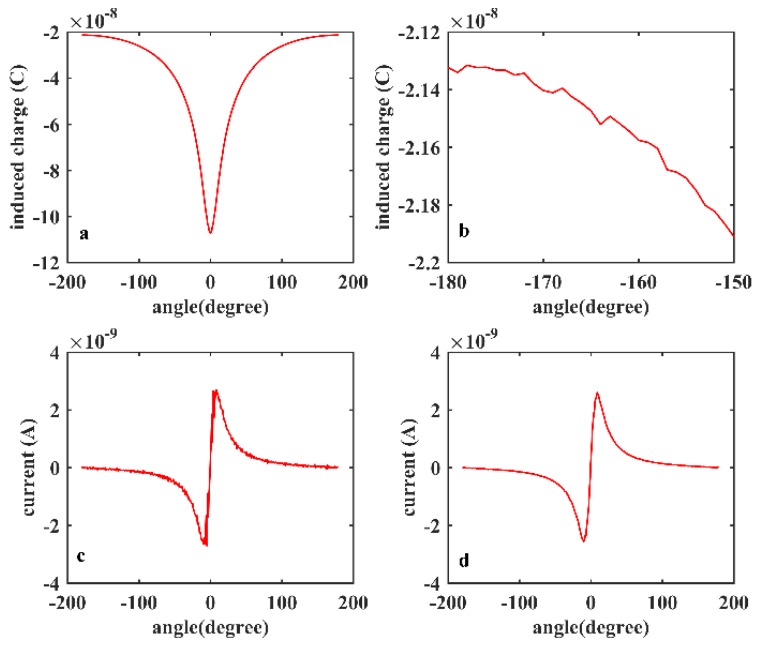
(**a**) Simulation result of induced charges; and (**b**) partial drawn of the induced charge. (**c**) Derivation of the induced charge; and (**d**) smoothed curve of the current.

Figure 8a depicts the induced charge on the electrode when the rotation of the strip begins farthest from the electrode, then passes by the electrode, and finally returns to the beginning position. The amount of induced charge on the electrode reflects the ‘far-near-far’ rotational process. The variation of charges on the electrode generates current *i*, which can be calculated by:

(14) $$i=\frac{dq_{e}}{dt}$$

where *q_e_* represents the amount of induced charge on the electrode. By calculating the difference of the induced charges, the current can be obtained and is shown in Figure 8c. When the strip is far from the electrode, the current is very weak and can be regarded as 0. When the strip rotates adjacent to the electrode, the current becomes larger. As seen from Figure 8c, the derivation of the induced charge contains thorns and wobbles. This can be explained due to the discretization and unavoidable computation error of the finite element simulation, the curve of induced charge is not smooth enough (shown in Figure 8b), thus leading to the thorns of the derivative curve. To acquire a more optimal result a moving average is applied to smooth the data and the result is shown in Figure 8d.

**Figure 9:**
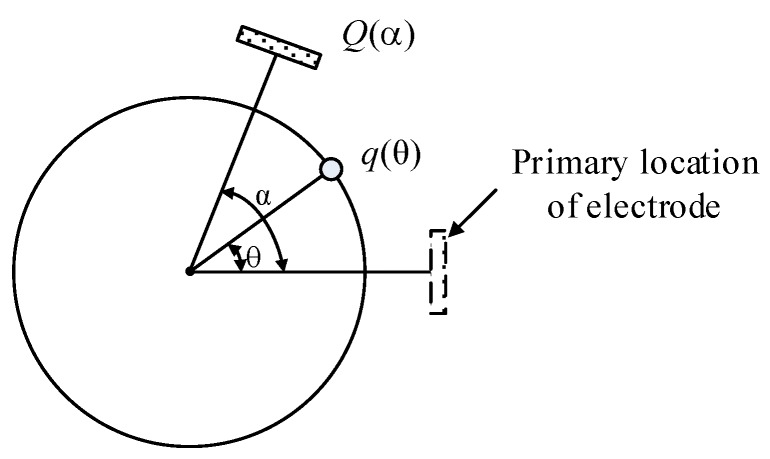
Schematic of electrode rotation.

Then, we use the rotation of the electrode to replace the rotation of shaft, so that the shaft is relatively at rest, as shown in Figure 9. According to the superposition principle of the electric field, the amount of induced charge *Q* on the electrode with a displacement of angle *α* can be calculated by Equation (15). Function *q*(*θ*) is the amount of charges on the location of angle *θ*. Parameter α indicates the rotated angle and is also a reflection of time. Function *f*(*θ−α*) means when a unit charge is *θ−α* degrees away from the electrode, the amount of induced charge on the electrode generated by this unit charge is *f*(*θ−α*). Thus, the total amount of induced charge can be calculated by integrating over *θ* from −180° to 180°.

(15) $$Q(\alpha )=\int_{-180{}^{\circ}}^{180{}^{\circ}}q(\theta )f(\theta -\alpha )d\theta$$

(16) $$Q(\alpha )=\int_{-\infty }^{+\infty }q_{circle}(\theta )f_{r}(\theta -\alpha )d\theta$$

**Figure 10:**
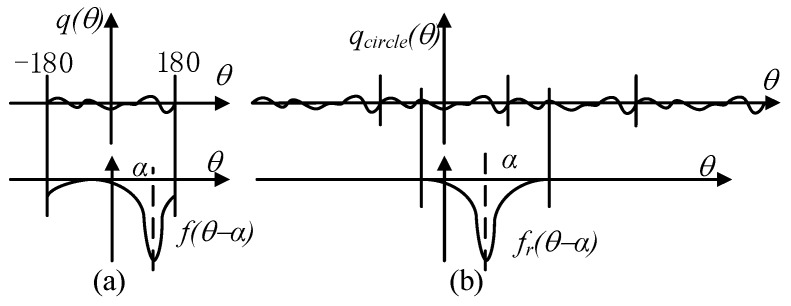
Explanation of Equations (15) (**a**), and Equations (16) (**b**).

As shown in Figure 10, Equation (15) illustrated by Figure 10a with an integral range of [−180° 180°] can be transformed into Equation (16) illustrated by Figure 10b. In Figure 10b, the values of *f_r_*(*θ*) when *θ* is out of range [−180, 180] are zeros and the waveform of function *f_r_*(*θ*) is the same as in Figure 8a, which is only different in amplitude. The induced charge on the electrode can be regarded as a weighted mean value of the contribution of the charge in a sensitive area. Meanwhile, Equation (16) is a convolution operation between the charge distribution function *q_circle_*(*θ*) and the function *f_r_*(*θ*), thus *f_r_*(*θ*) can be regarded as a filter function. The low pass filter property can also be obtained from [18]. Obviously, function *f_r_*(*θ*) is influenced by the rotational speed (*f_r_*(*θ*_0_ + *wt*)). The cutoff frequency of *f_r_*(*θ*) increases with the speed (narrow in the time domain, broad in the frequency domain). Through the analysis, the electrostatic electrode in this case of application can be regarded as a low pass filter which adaptively adjusts its cut-off frequency. Thus, the waveform of the signal mainly contains a low frequency component if the electro-magnetic interference is well shielded, which helps to explain the signal obtained in the experimental part.

## 3. Hardware Design

### 3.1. Sensor Board

**Figure 11:**
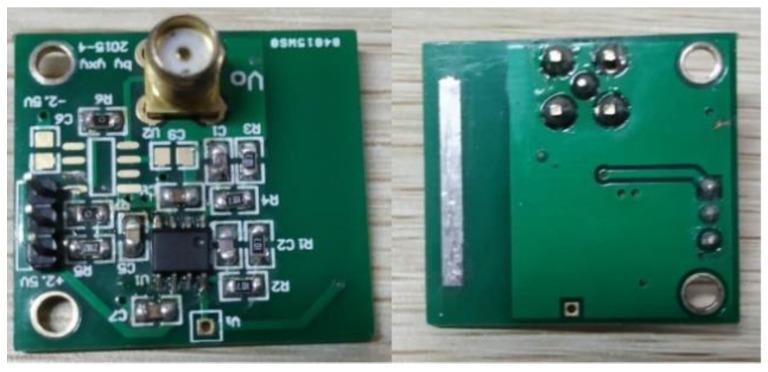
Sensor board.

**Figure 12:**
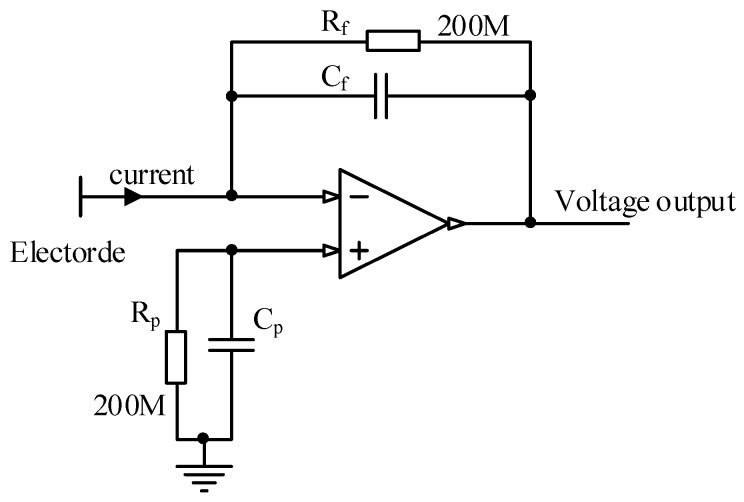
Schematic of current-to-voltage circuit.

The sensor board shown in Figure 11 contains an electrode and a current-to-current to voltage conversion circuit. A surface-tinned copper strip 20 mm long and 2 mm wide is utilized as the electrode. The electrode is connected to the current voltage conversion circuit, which is built on an LMP7721 (Texas Instruments in Dallas, TX, USA) amplifier with an extremely low bias current of 20 fA maximum. Figure 12 illustrates the schematic of the circuit. The feedback resistor consists of two 10^8^ Ω resistors connected in series, which determines the transimpedance gain. In actual application, a feedback capacitor is needed to guarantee the stability of the circuit by inhibiting the high frequency noise.

The relationship between the output voltage and the input current from the electrode can be calculated according to Equation (17). Thus, the sensitivity of the circuit is 0.2 V/nA. The purpose of the balance resistor and capacitor is to make the impedance of the two inputs equal, thus, the bias current of the amplifier generates no additional offset voltage on the output.

(17) $$U_{o}=R_{f}i_{e}$$

When the electric field near the electrode varies with the rotation of the charged strip, a small current signal will be generated and transformed into a voltage signal via the feedback resistance on the amplifier. The voltage output of the sensor board is collected by the condition unit via a shielded cable to avoid electromagnetic interference in the space.

### 3.2. Signal Condition Unit

**Figure 13:**
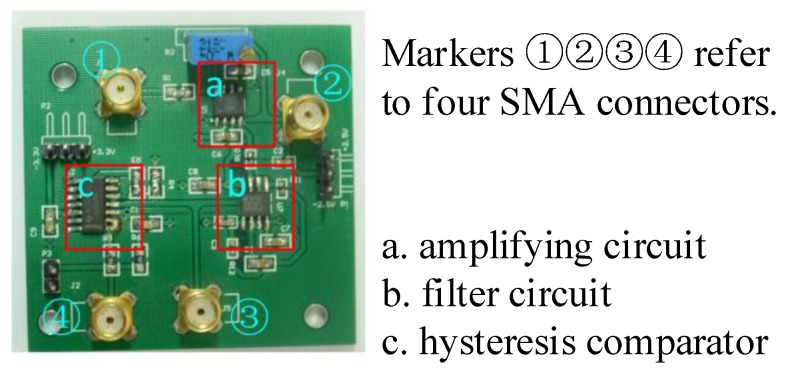
Signal condition unit.

The condition unit in our experiment is shown in Figure 13, which has amplifying, filtering, and comparing circuits designed to generate a square wave. Four connecters are placed on the board. Connecter 1 is used to connect to the output of the sensor board. Connecters 2 and 3 are used to observe the result of amplifying and filtering correspondingly. The square waveform from the comparing circuit is transmitted to a DSP chip via connecter 4 or the pin headers nearby.

The amplifying circuit uses the same amplifier chip with the sensor board to meet the performance requirements. The voltage gain of the amplifier can be adjusted by a slide rheostat. Then a third-order Butterworth low pass filter with Sallen-Key topology is used for filtering and inhibiting the noise. The passband frequency is 400 Hz and the stopband frequency is 2.4 kHz. A smooth waveform improves the stability of the square wave. Finally, a hysteresis comparator is utilized to transform the waveform into a square wave which is connected to the DSP board for speed calculation.

## 4. Experiment Results and Discussion

### 4.1. Experiment Conditions

**Figure 14:**
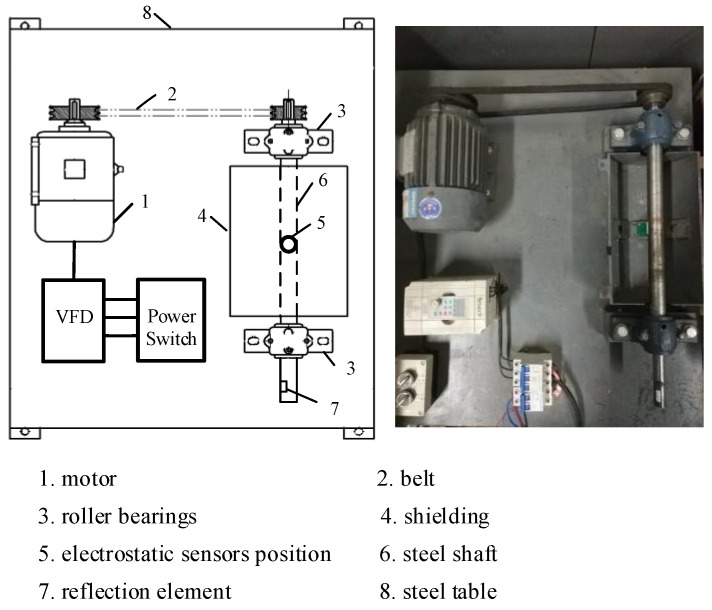
Schematic and photograph of the test rig.

**Figure 15:**
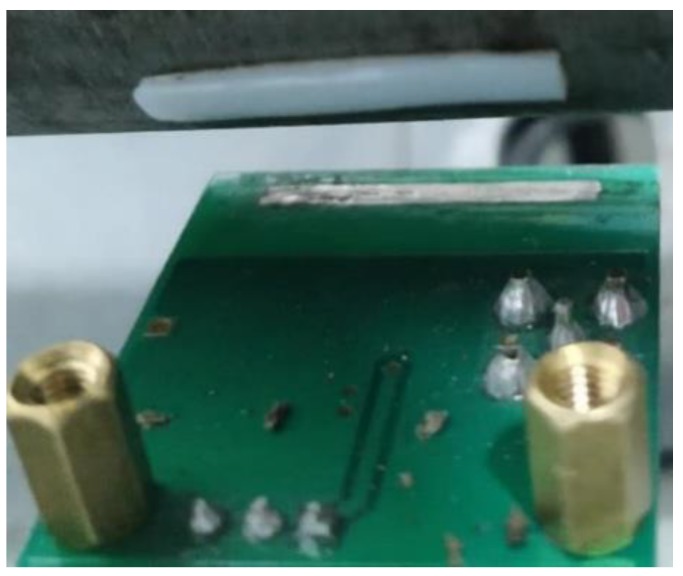
Photograph of the sensor and strip.

A laboratory-scale test rig is designed and built for rotational speed measurement. Figure 14 shows the schematic of the test rig. An external power supply connects to a variable-frequency drive (VFD) via a power switch. The torque of the motor is translated to the shaft via a belt. Thus, the rotational speed of the shaft can be adjusted by the VFD. The shaft is made of steel and supported by two roller bearings with a belt pulley mounted on its side. The middle part of the shaft is surrounded by a grounded cylindrical metal shielding. As shown in Figure 15, a strip of PTFE about 2 mm wide and 20 mm long, the lengthwise direction of which is parallel to the axial direction of shaft, is glued tightly on the shaft. The sensor board is set on the inner wall of metal shielding via a copper pillar, thus the electrode is under the central axis of the shaft and the trajectory of the strip. The copper pillar is utilized to adjust the distance between the sensor and the shaft. In order to inhibit the vibration of the rig, the steel table was screwed to the ground via an expansion screw.

Experiments were conducted on the rig using the same dimension parameters as the simulation. The rotational speed of shaft was adjusted from 300 rpm to 3200 rpm with an increment of 100 rpm via the VFD. To make a comparison between the digital approach and the correlation method, each point was measured five times. Meanwhile, five values of the *T* method and *M/T* method transformed from the DSP for each point were saved for analysis. A photoelectric reflection digital tachometer with an accuracy of ±0.05% of the reading plus 1 rpm was used to provide a reference speed in our experiment. The ambient temperature was controlled between 20 °C and 24 °C and the relative humidity was kept between 55% and 65%. The square wave was connected to the external interrupt pin to contend with the square wave immediately. The code of realizing the *T* method and the *M/T* method were programed and written into the DSP board separately to test the measurement performance. The DSP transmitted the measurement results of the *T* method and the *M/T* method to computer via RS232 serial communication. In the experiment, the prescribed time of the *M/T* method is set to be 1 s.

Seen from the principle of the correlation method, it can be found that the auto-correlation method can be regarded as a particular case of the cross-correlation method, which leaves out the influence of the installation angle error, the distance differences of the two electrodes to the shaft, and the differences between two channels’ circuits. These factors make the accuracy of cross-correlation method not as good as the auto-correlation method. Meanwhile, the cross-correlation method needs two channels of circuits, which is not consistent with the setting in this experiment. Thus, the experiment only makes a comparison between digital approaches and the auto-correlation method.

### 4.2. Signals

**Figure 16:**
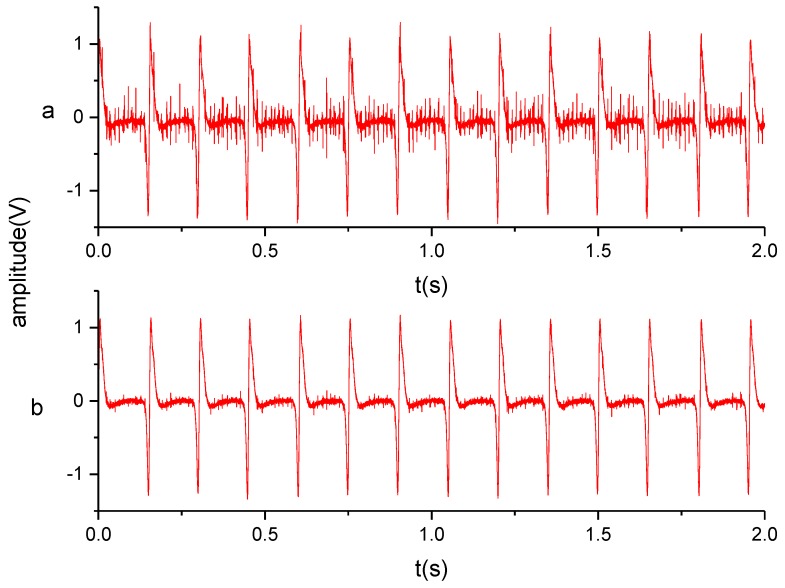
Input (**a**) and output (**b**) of the filtering circuit.

**Figure 17:**
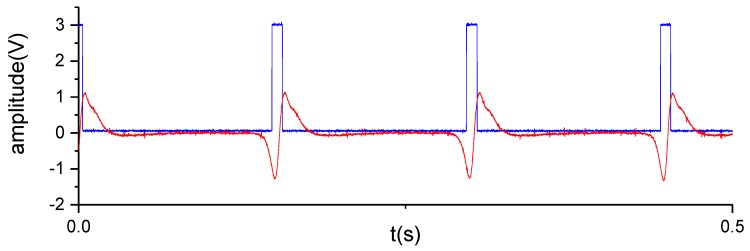
Analog input and digital output of the comparator circuit.

The proposed approach utilizes the electrostatic sensor to induce the charge on the strip of PTFE, which obtains a strong periodic signal. Figure 16 shows the signals before and after filtering, which contain evenly distributed waveforms similar to the simulation result (Figure 8d). The filtered waveform obviously has a higher signal-to-noise ratio. The high signal-to-noise ratio and the strong periodicity helps to improve the stability of the square wave transformed from the signal, which is very important for the rotational speed calculation based on the square wave. Figure 17 shows the square waveform generated by the hysteresis comparator. In order to illustrate the wave clearly, Figure 17 only shows 0.5 s of the signal.

### 4.3. Accuracy

**Figure 18:**
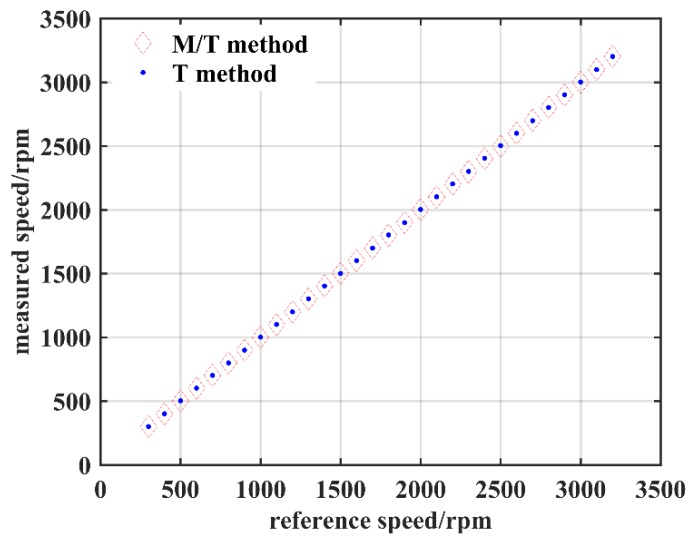
Measurement speed.

The mean values of the measurement results for the *T* method and the *M/T* method are plotted in Figure 18. Their relative errors are compared with the photoelectric reflection digital tachometer and are listed in Table 1. The linearity of the *T* method and *M/T* method are about 0.81‰ and 1.31‰, correspondingly. The measurement results are highly consistent with those of the photoelectric tachometer. Meanwhile, the differences between the *T* method and the *M/T* method are hardly discernible by eye.

**Figure 19:**
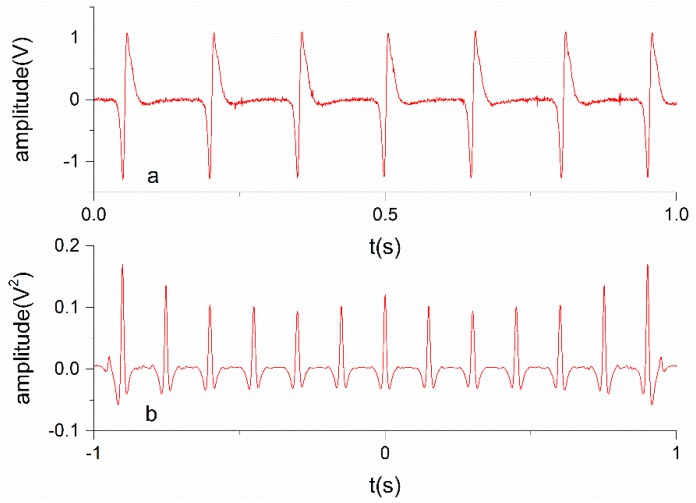
(**a**) Signal collected at the rotational speed of 400 rpm. (**b**) Auto-correlation of the signal.

As seen from the principle, the proposed digital method needs no sampling via an analog-digital converter, while the sampling rate is an important factor that determines the accuracy of the method based on the correlation algorithm. In order to make a comparison between these two methods, the analog signals are also collected at different sampling rates. The auto-correlation functions are calculated using the filtered analog signal. Figure 19 shows the auto-correlation of an example collected at the rotational speed of 400 rpm using a sampling rate of 2 ksps.

**Figure 20:**
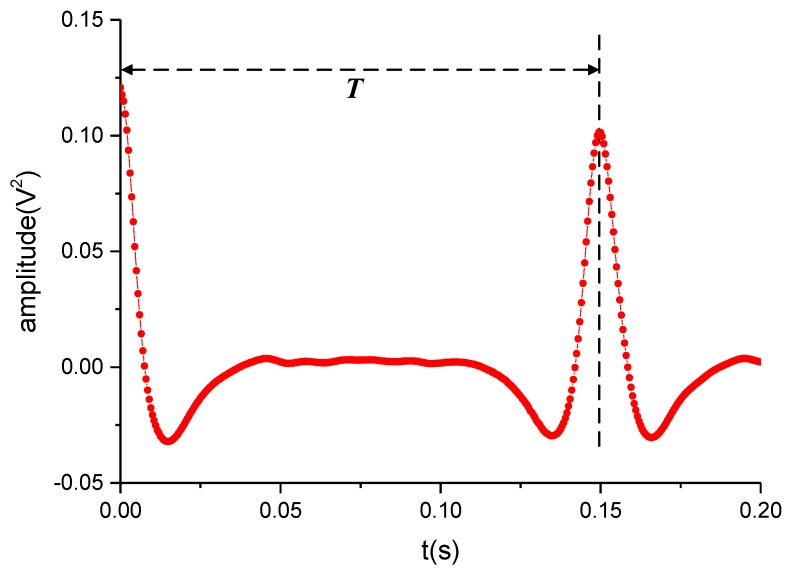
Partial enlarged drawing of Figure 17b.

The waveform in Figure 20 is a partial drawing of the part in Figure 19b. As shown in Figure 20, by detecting the first peak after 0 s, the period *T* of the rotation can be obtained. It can be observed that the waveform near the first peak after 0 s is very smooth, which benefits confirming the accurate and stable value of the period. However, due to the discretization of the data series, the obtained period *T* will be a time length away from the actual time of the rotation period with a significant probability despite the auto-correlation method confirming the nearest time point to the ideal time point. Moreover, when the signal contains an obvious level of noise or a weak periodicity, the waveform near the peak of the auto-correlation function will be fluctuant, which impairs the result’s accuracy.

**Figure 21:**
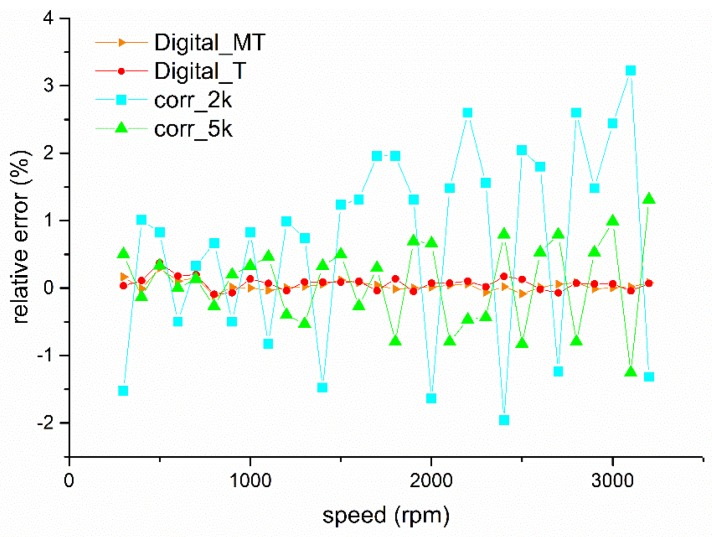
The absolute value of the relative error.

In order to show the accuracy of this digital method, signals are collected at the sampling rate of 2 ksps and 5 ksps and analyzed using the auto-correlation method. Relative errors of the two methods are plotted and compared in Figure 21. Figure 21 shows the measurement errors of the digital approaches and the auto-correlation method at the sampling rate of 2 ksps and 5 ksps. It can be seen from Figure 21 that the digital approaches have better accuracy and the relative errors obtained using 5 ksps are smaller than those sampled at 2 ksps. The auto-correlation method is apparently influenced by the sampling rate. Meanwhile, the accuracy of auto-correlation method has the tendency to increase with the rotational speed, which has been explained in 14. The linearity of the auto-correlation method sampled at 2 ksps and 5 ksps are 3.17% and 1.33%, respectively, which are significantly greater than those of the *M/T* method and the *T* method.

### 4.4. Standard Deviation

In order to research the robustness of the proposed method, the standard deviations of each measurement point are listed in Table 2. As seen from the table, regarding digital approaches, the *M/T* and *T* methods both have significantly small standard deviations. The standard deviations of the auto-correlation method in Table 2 contains a number of zeros and some other values, which can be easily understood from the principle. For example, using 5 ksps (Δt is 0.2 ms) to collect the signal of 1400 rpm, the period of which is about 214 times that of Δt, the calculated rotational speed by finding the first peak of the auto-correlation function will be some discrete value calculated by 60/[(214 ± n)Δt] (n = 0, 1, 2, …), like 1395.35, 1401.87, or 1408.45. There are two factors affecting the standard deviations of the auto-correlation method: (1) If the variation of rotation speed is not obvious enough to change the location of the first peak on the auto-correlation function, the measurement results will remain unchanged; and (2) if the locations of the first peak in the auto-correlation function differs one or two sampling intervals from each other due to signal differences, the obtained rotational speeds will show obvious fluctuations.

The standard deviations of the *M/T* and *T* methods in Table 2 are all within 1 rpm. Meanwhile, the standard deviations of the *M/T* method are much smaller than those of the *T* method. As seen from the principle, the *M/T* method can be regarded as a mean value of several consecutive *T* methods. Due to the high response speed of the *T* method, it is more sensitive to the variation of rotation, which makes its standard deviations greater than those of the *M/T* method. The minor standard deviations of the *M/T* and *T* methods mainly arise from the slight fluctuations of the actual rotation state, which is probably related to the unsteady output rotational speed of the motor and the slippage of the belt on the sheave. With respect to the digital approaches, no matter the *M/T* method or *T* method, both have very little spread in the measured speed.

### 4.5. Response Time

The response time of each approach can be determined from their principles and data process procedures. Regarding the *M/T* method, the response time is decided by adding extra time to the prescribed time. Regarding the analog method, usually the sampling length should be predetermined. Thus, the time needed to acquire one measurement result is nearly confirmed. Even if the auto-correlation method self-adaptively adjusts the sampling length according to the nearest obtained rotational speed, the response speed is still not as fast as the *T* method for the reason that the auto-correlation method needs at least two periods of rotation to achieve the correlation calculation. Moreover, data collection and processing also consume a certain amount of time.

**Figure 22:**
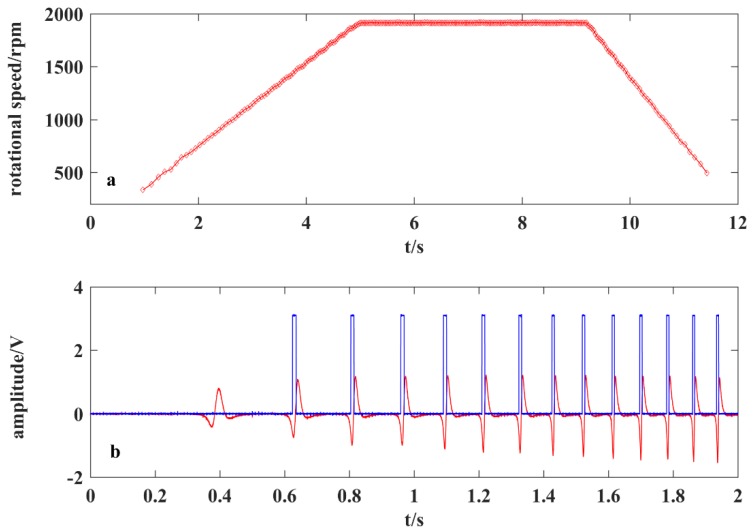
(**a**) Measured rotational speed by the *T* method; and (**b**) waveform during acceleration process.

By contrast, the *T* method can output a result every rotational period for the reason that the counter in the DSP can work independently from the code and the DSP only needs to perform an easy computation of the counter number and serial communication. Experiments were conducted to test the capability of the *T* method to measure the variable speed. The motor was adjusted by the VFD output frequency to work at three stages: acceleration by increasing the frequency from 0 Hz to 20 Hz over 5 s, 4 s of constant speed, and deceleration by decreasing the frequency from 20 Hz to 0 Hz over 3 s. Figure 22a shows the 256 acquired rotational speeds via the *T* method, which successfully monitors the acceleration and deceleration processes. The rising and decline curves are not perfectly straight lines because the acceleration of the shaft is not absolutely constant. It can be observed that at a constant frequency, the measurement results are of good stability.

Figure 22b shows a part of the waveform during the acceleration stage. The acceleration and deceleration process can be clearly observed through the interval variations of square waves. The waveform near 0.5 s has not been transformed into a square wave because a limited electrostatic charge is generated on the strip at low speed, thus making the signal unsuitable for the following square wave generation circuit. With the increases of rotational speed, the amount of charge rises and then becomes stable because of the dynamic balance reached between the natural discharge and recharge. The signal amplitude changes as the unbalanced charge increases or decreases at a low speed, which makes the comparison voltage appear at different positions relative to the waveform. This phenomena limits the application of this method in measuring low variable speed.

## 5. Conclusions

The work in this paper dedicates to find a more effective approach to cooperate with electrostatic sensors to improve the performance of rotational speed measurement. The proposed approach utilized the electrostatic sensor to induce a charge on a strip PTFE, which obtained a strong periodical signal. Simulation results also described the expected waveform when a strip of charges rotates near an electrode. By adopting a suitable signal condition unit, a square wave, the frequency of which was equal to that of the rotational speed, has been obtained. Having the square wave proportional to rotational speed, the *M/T* method and *T* method were adopted to calculate the speed in a DSP system. Experiments were conducted to compare the digital approaches with the auto-correlation method. Through experimental analysis, several conclusions can be summarized as follows:

1. Accuracy: Compared with the auto-correlation method, the *M/T* method and *T* method both have an obviously higher accuracy. The linearity of the *M/T* method and *T* method are about 0.81‰ and 1.31‰, correspondingly, which are much better than those of the auto-correlation method sampled at 2 ksps (3.17%) or 5 ksps (1.33%). Due to the signal discretization, the auto-correlation method can only obtain some discrete values. Improving the sampling rate, calculation quantity, and storage space, the hardware cost will also increase correspondingly.

2. Robustness: The auto-correlation method has a stable performance in some measurement points and also has some obvious standard deviations, which resulted from the signal discretization. However, the *M/T* method and *T* method obtained particularly small standard deviations among all the measurement points, both within 1 rpm. The *M/T* method acquired more stable results than the *T* method due to differences of their respective principles.

3. Response speed: The proposed approach combined with the *T* method has the fastest response speed. The correlation method and *M/T* method have relatively slower response speeds. Experiments also shows that the *T* method is capable of detecting the variable speed.

Indeed, having the square wave related to the rotational period, the *M/T* method can be adopted for constant speed measurement or a mean value of rotational speed during a certain time and the *T* method can be employed for dynamic measurement of variable rotational speed. In actual programming, the *M/T* method and *T* method can be written into one piece of a DSP or FPGA simultaneously. An FPGA is more recommended to deal with the square wave for its property of parallel processing and high code execution efficiency.

There are several factors limit the application of this method working at a low speed. The amount of charge on the strip is unstable and the response time is poor at low speed. Further studies can be conducted to deal with these issues by adopting an electret material, adding adaptive numbers of strips and electrodes, and improving circuit properties.

## Figures and Tables

**Table 1:** The relative errors of the measurement points.

Reference Speed (rpm)	Measured Speed (rpm)		Relative Error (‰)	
	*M/T*	*T*	*M/T*	*T*
300	300.50	300.11	1.67	0.36
400	399.97	400.45	−0.08	1.12
500	501.04	501.87	2.08	3.74
600	600.63	601.08	1.05	1.80
700	701.09	701.39	1.56	1.98
800	799.12	799.26	−1.10	−0.92
900	900.10	899.38	0.11	−0.69
1000	1000.02	1001.35	0.02	1.35
1100	1099.65	1100.79	−0.32	0.72
1200	1200.06	1199.55	0.05	−0.38
1300	1300.30	1301.22	0.23	0.94
1400	1400.83	1401.27	0.59	0.91
1500	1501.81	1501.34	1.21	0.89
1600	1601.33	1601.60	0.83	0.99
1700	1700.71	1699.35	0.42	−0.38
1800	1799.75	1802.47	−0.14	1.37
1900	1900.12	1899.11	0.06	−0.47
2000	2000.34	2001.50	0.17	0.75
2100	2100.87	2101.57	0.41	0.75
2200	2201.38	2202.34	0.63	1.06
2300	2298.58	2300.50	−0.62	0.21
2400	2400.52	2404.21	0.22	1.75
2500	2497.95	2503.25	−0.82	1.30
2600	2600.32	2599.51	0.12	0.19
2700	2701.59	2698.14	0.59	−0.69
2800	2802.13	2802.10	0.76	0.75
2900	2899.71	2901.81	−0.10	0.62
3000	3000.15	3001.87	0.05	0.62
3100	3100.52	3098.77	0.17	−0.40
3200	3202.58	3202.23	0.81	0.70

**Table 2:** The standard deviations of the measurement points.

Reference Speed (rpm)	Standard Deviation (rpm)			
	*M/T*	*T*	2k	5k
300	0.055	0.175	1.755	2.047
400	0.013	0.292	2.641	2.250
500	0.013	0.397	4.652	1.867
600	0.032	0.274	1.651	2.161
700	0.023	0.458	3.437	1.472
800	0.097	0.142	2.367	2.592
900	0.091	0.115	3.011	1.206
1000	0.068	0.378	0	2.790
1100	0.011	0.146	0	0
1200	0.044	0.190	0	0
1300	0.015	0.284	0	2.525
1400	0.051	0.038	0	0
1500	0.010	0.027	0	0
1600	0.013	0.265	0	4.674
1700	0.017	0.190	0	0
1800	0.019	0.467	0	2.542
1900	0.010	0.065	0	0
2000	0.027	0.284	0	0
2100	0.029	0.234	0	0
2200	0.051	0.006	18.069	0
2300	0.016	0.368	0	7.878
2400	0.051	0.281	0	0
2500	0.050	0.415	0	0
2600	0.048	0.155	0	0
2700	0.018	0.364	0	0
2800	0.069	0.082	0	0
2900	0.039	0.301	0	0
3000	0.052	0.131	0	0
3100	0.012	0.327	0	0
3200	0.094	0.345	46.747	0
